# Tailormade PMMA Spheres: Synthesis and Growth Mechanism

**DOI:** 10.1021/acsomega.5c00402

**Published:** 2025-06-04

**Authors:** Oliver Thüringer, Raphaell Moreira, Marcus Bäumer, Cecilia B. Mendive, Thorsten M. Gesing, Alexander Wollbrink

**Affiliations:** † Institute of Inorganic Chemistry and Crystallography, 9168 University of Bremen, Leobener Straße 7, 28359 Bremen, Germany; ‡ Institute of Applied and Physical Chemistry, University of Bremen, Leobener Straße 6, 28359 Bremen, Germany; § MAPEX Center for Materials and Processes, University of Bremen, Bibliothekstraße 1, 28359 Bremen, Germany; ∥ Facultad de Ciencias Exactas y Naturales, Departamento de Química, 28233 Universidad Nacional de Mar del Plata, B7602AYL Mar del Plata, Funes 3350, Argentina

## Abstract

Inverse opal structures are of interest for various applications, as they exhibit high surface areas in conjunction with unique structure-specific properties such as the possibility to create photonic band gaps, e.g., for photocatalytic applications. An established synthetic pathway to prepare these nanostructures is to infiltrate the voids of a template comprised of close-packed spheres with a metal oxide and to remove the template subsequently by pyrolysis. To this end, polymer spheres are typically used which are produced by a water-based emulsion polymerization process. In this work, we present an improved and extended approach of that kind in case of PMMA spheres—featuring narrow size distributions and mean diameters that can be varied over a large range between 170 up to 800 nm by properly adjusting the synthesis temperature and the ionic-strength of the water phase. By using reflux conditions, advanced experimental techniques requiring protective gas atmospheres are dispensable and comparatively short synthesis times can be realized. Time-resolved experiments reveal a two-step growth process occurring at temperatures below ∼400 K. It consists of a first phase, during which initial particles are formed, followed by a time-delayed second phase, where their diameter increases by roughly a factor of 2, most likely due to coalescence processes. At higher temperatures, both processes increasingly overlap so that only a single growth phase is observed.

## Graphical abstract

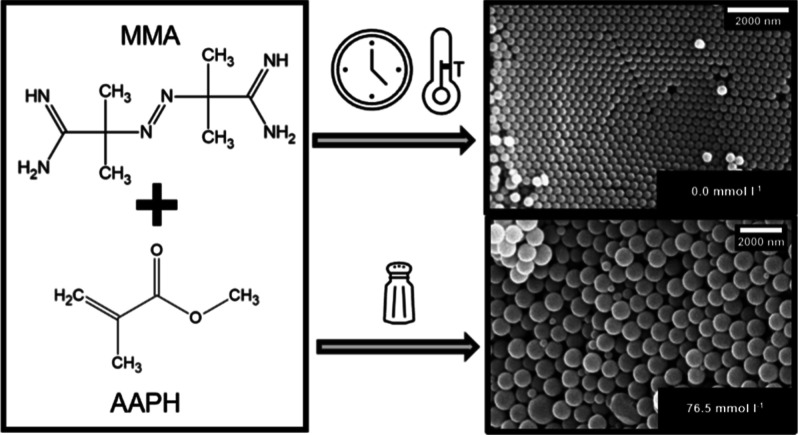

## 1 Introduction

Inverse opals represent a particularly interesting class of well-ordered nanostructures.^1,2^ Not only they exhibit high surface areas,^3^ but their regularly arranged spherical void volumes entail photonic properties that render them attractive for various optical applications.^4^ In case of photocatalytic reactions, for instance, a proper adjustment of their photonic band gaps allows for enhancing the catalytic efficiency considerably.^5^ A usual synthetic approach to produce inverse opal systems is to infiltrate the voids of a template structure consisting of closed-packed spheres (diameter typically in a range from a few nano- to micrometers^6^) in a spatially extended and periodic arrangement.^7^ To this end, the latter obviously need to exhibit a uniform size or, in other words, their size distribution needs to be as narrow as possible.

Since the template is typically removed subsequently by pyrolysis, a natural choice are polymer spheres. Classes of polymers frequently employed for this purpose are for example polyaniline,^8^ polyvinyl chloride,^9^ polystyrene^10,11^ or polymethylmethacrylate (PMMA),^12,13^ among which the latter (PMMA) is the most harmless one and, for this reason, used in many other applications, too.^14^ An established way to prepare such spheres is a procedure called emulsion polymerization process, based on a radical chain reaction.^15^ To this end, the corresponding monomer and a suitable surfactant are mixed with water initially. Upon intensive stirring, an emulsion of monomer droplets distributed in the water phase and stabilized by the surfactant is formed then. Even if the monomer is hardly soluble in water, a certain fraction is nonetheless expected to remain in the aqueous environment. These dissolved monomers will immediately polymerize to oligomers, when an initiator is added starting the radical chain reaction. Since the surfactant will not only stabilize the droplets but—if added in excess—will also form micelles in the water phase, it plays a role in the subsequent growth process by taking up the (apolar) polymer chains and encapsulating them. Within these compartments, the oligomers can then continously increase in length by attaching additional monomers, which are successively incorporated into the micelles.^15^ The termination of this process and hence the size of the polymer spheres finally obtained depend on the temperature as well as on the ratio of surfactant and monomer and can—on this basis—be experimentally controlled.^16^

Being still adsorbed on the surface of the particles at the end of the synthesis, the surfactant provides the advantage of preventing their agglomeration in the final colloidal dispersion. At the same time; however, it bears the disadvantage of hindering the formation of well-ordered closed-packed layers when coating the spheres on a surface and drying them.^12^

When using monomers, such as, e.g., MMA, which are better soluble in water, the use of a surfactant is dispensable and a so-called surfactant-free polymerization becomes alternatively possible.^15^ The oligomers that are formed in the water phase upon adding the initiator do not need a surfactant or corresponding micelles to grow. They successively aggregate to first seed particles which then further grow by attachment of monomers diffusing in the emulsion. Actually, the initiator radicals take the role of a stabilizer here. By terminating the polymer chains at the surface of the particles, they prevent their agglomeration at the end of the polymerization process—in analogy to a surfactant.^15^ Being less bulky; however, this situation is less detrimental in regard to the arrangement of the spheres to well-organized close-packed layers, when intending to use them as templates for inverse opal structures, for instance.

While accordingly the surfactant-free pathway provides some advantages, both of these well-established approaches feature common synthetic disadvantages, such as the need to exclude molecular oxygen (interfering, as a biradical, with the radical chain reaction)^17^ and comparatively long preparation times.^18^ In 2007, Gu et al.^17^ proposed an alternative for the surfactant-free fabrication of PMMA spheres, avoiding such complications. By using reflux conditions, a protective gas atmosphere was dispensable and, due to the higher temperatures, synthesis times could be significantly shortened. As only a limited temperature window (from 353 to 373 K) was tried by these authors, though, only spheres within a relatively narrow size regime between 250 and 300 nm were accessible.

Inspired by this work, we have explored this interesting synthetic pathway further and not only extended the corresponding temperature range but also investigated the influence of the ionic strength within the water phase on the range of obtainable sphere sizes. Egen et al.^12^ as well as Tanrisever et al.^13^ have already noticed that an increase in ionic strength results in larger sphere diameters, but studied this effect only for salt concentrations up to 30 mmol·L^–1^. Our aim was to extend this range and to check the potential of this parameter to control the growth under reflux conditions. Furthermore, we carried out time-resolved experiments at different temperatures to understand the mechanistic background of the underlying formation process in more detail. Finally, we characterized the opalescence properties by diffuse reflectance UV/vis spectroscopy to verify the suitability of the layers, obtained after coating and drying the particle suspensions on a planar surface, as templates for inverse opal nanostructures. The results are presented in three separate subsections of Section 3.

## 2 Experimental Section

### 2.1 Synthesis

For all reactions the solvents and chemicals employed exhibited “ACS grade” purity: methyl methacrylate (MMA, VWR, 99.5%, stabilized with 10–25 ppm 4-Methoxyphenol), 2,2′-azobis­(2-amidinopropane) dihydrochloride (AAPH, Acros Organics, 98% purity) and sodium chloride (NaCl, VWR chemicals, 100%).

#### 2.1.1 Temperature-Dependent Reactions

PMMA spheres were synthesized using a batch emulsion polymerization process. To this end, a 250 mL three-neck round-bottom flask was equipped with a reflux condenser, a magnetic stirrer and a temperature-controlled heat-on-block heater (temperature accuracy: about 1 K). For the reaction carried out under continuous stirring (5 Hz), 10 mL of MMA were added to 80 mL demineralized water according to a volumetric ratio of MMA/H_2_O = 1:8. The reaction temperatures were varied in the range between 363 and 423 K in 10 K steps, using a constant synthesis time of 120 min. Upon reaching the selected temperature, in all cases 0.075 g (0.27 mmol) 2,2′-azobis­(2-amidinopropane) dihydrochloride as a water soluble initiator were added to the emulsion. After some minutes (see Section 3) a color change of the reaction mixture from greyish to white could be observed. Following the synthesis, all samples were dried at room temperature for approximately 48 h, without any additional processing.

#### 2.1.2 Time-Dependent Reactions at Selected Temperatures

To get further information about the reaction progress, time-dependent experiments were performed at selected temperatures (363, 393 and 433 K). Initiating the reactions as described above, 0.5 mL of the reaction volume were removed each minute from the emulsion within an time interval between 3 and 20 min after adding the initiator. The samples were directly cooled down in a fridge to 276 K to stop the reaction and then dried on a metal holder under ambient conditions. Boltzmann-sigmoids were used to fit the data.

#### 2.1.3 Ionic Strength Dependency

To test the influence of the ionic strength within the water phase previously shown to influence the sphere diameter,^12,13^ selected amounts of sodium chloride were added to the prepared MMA-water emulsion, resulting in concentrations in the range between 19.2 and 76.5 mmol·L^–1^ (with an error of about 1.0 mmol·L^–1^). Afterwards, the mixture was heated to 383 K and the initiator added in the same way as before. All syntheses were again carried out employing a total reaction time of 120 min. To avoid salt inclusions in the PMMA spheres, the dried PMMA spheres were additionally washed three times with about 30 mL of demineralized water and centrifugated for separation. Subsequently, the PMMA spheres were again dried at ambient conditions.

### 2.2 Characterization

#### 2.2.1 Scanning Electron Microscopy (SEM)

SEM micrographs were acquired with a Jeol JSM-6510 (JEOL GmbH, Munich, Germany) instrument. Using an acceleration energy of 20 kV, a spot size of 30 (in a range of 0–100) was selected at a working distance of 10 mm. Energy-dispersive X-ray backscattering (EDX) spectra were recorded within an energy range from 0.1 to 20 kV, using the implemented Bruker X-M 410 detector which was operated with the Bruker Esprit 1.9 software. To avoid surface charging, the spheres were placed on a carbon glue tape fixed on an aluminum sample holder and sputtered with Au in a reduced argon atmosphere under a pressure of about 8 Pa.

The average sphere diameter (ASD) of selected PMMA spheres was quantitatively determined on the basis of SEM micrographs, using the open access software Fiji.^19^ To this end, at least 60 spheres per sample were evaluated. In terms of the IUPAC definition, the ASD is equivalent to the so-called “number-average particle diameter”.^6^ The determined sphere-size distributions were fitted with a Gaussian distribution function, using the software Origin 2024b (version 10.1.5.132).

#### 2.2.2 UV/Vis Spectroscopy

Diffuse reflectance UV/vis spectroscopic measurements were carried out with a Shimadzu UV/vis spectrophotometer (UV-2600) that was equipped with an ISR-2600 plus two-detector integrating sphere and operated with the software UVProbe (Version 2.70). Barium sulfate was used as a 100% white reference standard. Spectra were recorded in the wavelength range between 190 and 850 nm in steps of 1 nm with a medium scan speed and a slit width of 5.0 nm. The obtained spectra were background corrected, using a polynomial approach, and the peaks were fitted with a Gaussian distribution function. Their full width at half-maximum (fwhm) was used to calculate the respective two-sigma standard deviations of the determined peak maximum (2σ).

## 3 Results and Discussion

### 3.1 Growth Mechanism

In the Introduction the generally accepted notion regarding the formation and evolution of polymer spheres by an emulsion (radical) polymerization process with and without a surfactant, as, e.g., described in the review of Lovell and Schork^15^ or in the work of Egen and Zentel,^12^ was briefly discussed.

When comparing the two cases, it is obvious that in the first one the surfactant plays a decisive role as structure-directing agent, since the growth process does not directly take place in the water phase but within micelles, the amphiphilic surfactant molecules form therein. In contrast, for the surfactant-free variant the relevant reaction steps immediately occur in the water phase and, due to the lack of such predefined reaction compartments, a more complex mechanistic situation can be expected here. Although well-established recipes can be found in the literature also in this case allowing to prepare well-defined polymer particles with desired diameters and narrow size distributions, the mechanistic understanding of the whole process and of all involved single steps is less profound, as compared to surfactant-assisted synthesis routes.

This is particularly true for a relatively new variant of the surfactant-free approach carried out under reflux conditions, as first proposed by Gu et al. for PMMA spheres.^17^ While opening an attractive, since experimentally less demanding preparative pathway for this class of polymer particles, neither details of the growth process nor its full potential to obtain uniform spheres over a large size range have been dealt with in this first work or in any later study.

For this reason, we conducted a series of time-dependent measurements to get deeper insight into the growth mechanism and its dependence on the temperature. Starting with 363 K, falling into the narrow window of low temperatures, Gu et al.^17^ already investigated (353–373 K), we extended the range to 433 K and included 393 K as an intermediate temperature in between these values. In each case small amounts of the reaction solution were sampled (and quenched) every minute (60 s) after adding the initiator. Subsequently, these dispersions were coated on a metal holder, dried and analyzed by SEM. On this basis, the diameters of the particles formed between reaction start and the respective sampling time could be determined and their average sphere diameter (ASD) evaluated (see Section 2). Figure 1 shows the evolution of the latter as a function of time for the three temperatures investigated.

**Figure 1:**
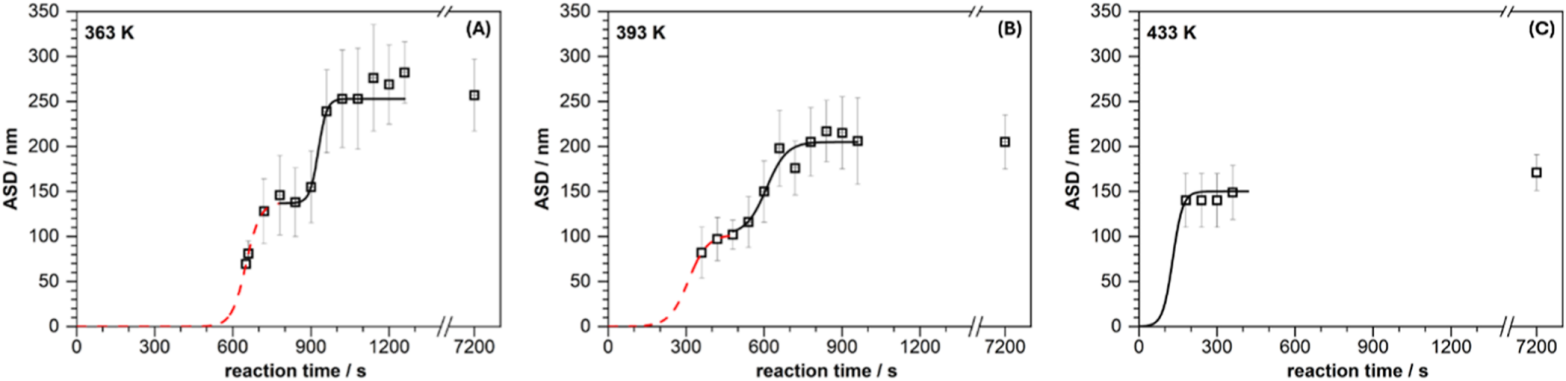
Arithmetic average sphere size (ASD) as a function of time for three reaction temperatures: 363 (A), 393 (B) and 433 K (C). The red dotted line in the first two cases indicates the first growth phase (formation of seed particles). The black lines denote the second phase attributed to the further growth by particle coalescence. The ASD values at the time of 7200 s (120 min) were taken from the corresponding temperature-dependent experiments (see Figure 2).

For the lowest temperature of 363 K the first spheres were detected after a reaction time of about 660 s which exhibited an ASD of about 80 nm. As inferable from Figure 1A, thereafter the ASD sharply increases at first (sphere growth rate: ∼0.9 nm/s) to reach, however, a plateau shortly after. Over a period of about 180 s the ASD do not vary and stay constant at a value of about 140 nm. After the plateau, a second sharp increase of the ASD is observed (sphere growth rate: ∼1.5 nm/s), leading, after ∼1000 s, to a final value of about 240 nm which does not change then anymore (within the error margin).

For the intermediate temperature of 393 K, qualitatively a similar behavior is revealed, as compared to 363 K (Figure 1B). Again, two growth steps—even though in this case the plateau is less pronounced as the steps partly overlap each other—can be discerned and the first spheres detected by SEM showed an ASD of ∼80 nm, in analogy to 363 K. In contrast to the latter, however, these particles emerged earlier, i.e., already after 360 s. This finding is of course not surprising in view of a faster polymerization rate to be expected at the higher temperature. A further difference refers to the plateau between the two growth steps which not only is shorter (roughly by a factor of 2) but also occurs at a lower ASD (∼110 nm). In addition, the rise in ASD in both stages is considerably less pronounced, i.e., less steep (sphere growth rate ≈ 0.5 nm/s in both instances) compared to the reaction at the 30 K lower reaction temperature. Overall, it seems that the phases being well-separated in the latter case merge and increasingly overlap when rising the reaction temperature.

This assumption is corroborated when including the highest reaction temperature (433 K). Here, only one growth phase can actually be identified. Already after ∼180 s, first spheres with an ASD of ∼140 nm were detectable. In contrast to the lower temperatures, this value only slightly increases in the following (to ∼170 nm), meaning that in this case an average diameter is early on reached which essentially corresponds to the final one.

As mentioned in the Introduction, it is important to not only consider ASDs but also the underlying (Gaussian) size distributions of the polymer particles. As shown in the Supporting Information (Table S1), these indeed turn out to be relatively narrow (2σ ≈ 20%) throughout the whole growth process in all three cases (363, 393 and 433 K)—rendering the PMMA spheres prepared in this way well-suited as templates for the fabrication of inverse opal nanostructures. We will come back to this point in the next subsection.

Even though their data did not allow drawing clear-cut conclusions in this regard, already Gu et al.^17^ speculated in their pioneering work on the synthesis of PMMA spheres under reflux conditions that the formation process might take place in two steps.^17^ During a first *chemical* growth phase “seed” particles are created by polymerization of monomers dissolved in the water phase to oligomers which subsequently agglomerate. It was suggested that this formation phase is followed by a second *physical* growth phase where coalescence or Ostwald ripening of the primary particles occurs,^15,20^ but the experimental data did not allow a distinct conclusion in this respect. Based on our results which clearly indicate a two-step process—at least in the lower part of the temperature window investigated—such a mechanistic scenario can indeed be confirmed. As far as the first step (red dotted line in Figure 1A,B) is concerned, Boltzmann sigmoids were used to fit the particle size data, in agreement with the Smith–Ewart model.^15^ The initial phase occurs before detectable particles appear, corresponding to micelle formation. The growth process is reflected by the slope, while the stagnation phase is represented by the upper plateau of the sigmoid. Provided that the oligomer chains within the seed particles formed in the first step are not yet cross-linked to rigid polymer entities, subsequent dynamic changes within the particle ensemble will be possible. The steep increase in size seen after the first plateau points to coalescence processes in this regard, finally resulting in PMMA spheres which exhibit a 5- (393 K) up to 6-fold (363 K) larger volume, as compared to the original seed particles. The finding that the latter are smaller (110 nm) at 393 K than at 363 K (140 nm) can be straightforwardly explained in this context by the faster decomposition of the initiator (Int–Int → 2 Int·) at the higher temperature. Since this step actually represents the rate-determining step of the whole polymerization reaction, the higher concentration of radical starters in the emulsion will result in a higher number of oligomer chains simultaneously growing. When these eventually agglomerate, more, but smaller seed particles will be generated—in agreement with the observation.

Coming finally to the highest reaction temperature studied, i.e., 433 K, the circumstance that in this case the growth of the PMMA particles seems to take place not in two but rather in one step can, in principle have two different causes. Either the chemical and physical growth processes increasingly overlap so that they eventually are no longer experimentally separable, or the higher temperature results in such high polymerization rates from the start that fast cross-linking in the primary seed particles efficiently prevents later coalescence among them.

At first glance, this question is not easily decidable on the basis of the present results. On the one hand, it is known that above ∼400 K the decomposition of the used initiator is noticeably accelerated,^21^ suggesting indeed distinctly higher polymerization rates in this temperature region and thus favoring the second assumption. On the other hand, however, the finding, that the two steps continuously merge with rising temperature (see Figure 1), would rather support the first explanation, also appearing plausible in view of the expected higher density of seed particles (corresponding to shorter interparticle distances on average) and the higher diffusion rates. It is also worth noting in this context that the ASD of ∼140 nm achieved at 433 K lies well above the size of the seed particles formed at 393 K (110 nm) in the first (chemical growth) stage. Since the diameter of these primary particles should further decline when moving from 393 to 433 K, it is unlikely that the 140 nm refer to them. A more likely scenario is a fast formation of small seed particles—smaller than at 393 K—which, at this higher temperature, coalesce more or less instantaneously into the larger spheres finally detected in the SEM images.

The temperature-dependent experiments which were additionally carried out provided further insight in this context. Here, the final ASDs, i.e., the sphere diameters obtained at the end of the whole growth process (reached in all cases within the total synthesis time of 2 h = 120 min), were determined for selected temperatures also lying in between those chosen for the time-dependent series. The corresponding results are presented in Figure 2. [The underlying numerical data are compiled in the Supporting Information (Table S2), where also SEM micrographs of PMMA spheres synthesized at 363, 383, 413 and 433 K as well as the respective size histograms can be found (Figure S1).]

**Figure 2:**
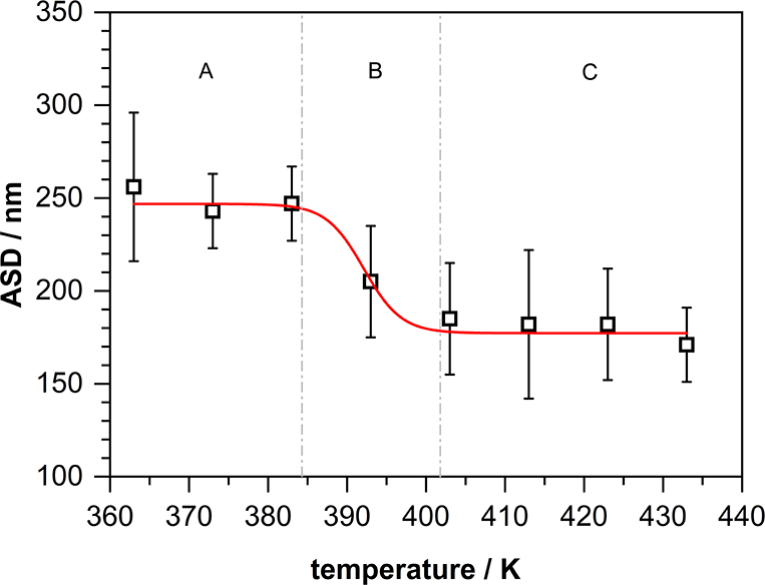
Arithmetic average sphere sizes (ASD) of PMMA spheres obtained between 363 and 433 K after a total synthesis time of 120 min (The red line represents a sigmoidal fit of the data); A, B and C indicate the three different temperature regimes discussed in the text.

Up to ∼383 K, the ASD of all particle ensembles is essentially constant and identical to the value observed already at 363 K (∼245 nm, see also Figure 1A). The same is true for temperatures above ∼403 K, where all determined ASDs correspond (within the error margins) to the value also seen for 433 K in the time dependent series (∼170 nm, Figure 1C). A change of the ASD (from the higher value at lower temperatures to the lower value at higher temperatures) is only recognized in a small temperature window lying between these two regimes (383–403 K), where, according to Figure 1B, the two-step particle formation descends to a one-step growth.

Noticeably, this window falls exactly in the range where the decomposition rate of the initiator is expected to speed up. In conjunction with the arguments given above, this agreement suggests the following temperature depended growth behavior:

(A) *T* < 383 K: slow initiator decomposition → growth of fewer seed particles with larger diameter → further growth by coalescence at a later stage ⇒ two-step process.
(B) Transition in the intermediate regime (383–403 K) ⇒ both phases start overlapping.
(C) *T* > 403 K: fast initiator decomposition → growth of significantly more seed particles exhibiting smaller diameter and concomitant coalescence to larger particles ⇒ one-step process.

### 3.2 Addition of NaCl: Variation of Sphere Sizes

The data presented in the previous subsection reveal that only a rather limited size range of PMMA spheres can be assessed by solely varying the synthesis temperature. In comparison to Gu et al.,^17^ who reported for 350–370 K diameters between 250 and 300 nm, our results indicate that also somewhat smaller ASDs down to 170 nm are assessable, when raising the synthesis temperature by about 60 K. Yet, for the reasons given above significant larger sizes are obviously not obtainable with the plain approach.

As mentioned in the Introduction, however, Egen and Zentel^12^ as well as Tanrisever et al.^13^ have shown for standard surfactant-free emulsion polymerization processes of PMMA spheres that another strategy to tune particle sizes is opened by increasing the ionic strength of the water phase. Upon adding ionic salts, such as alkali halides, for instance, a considerable extension of the size range toward larger diameters could be achieved.

To check this strategy in the present case, increasing concentrations of NaCl were added to the emulsion before starting the polymerization reaction. To this end, a synthesis temperature 383 K was chosen. As revealed in Figure 2 and detailed in the previous subsection, this value lies at the upper end of the lower temperature window, where chemical (seed particle formation) and physical growth (coalescence) are still clearly separated.

The results are shown in Figure 3 (size distributions) and Table 1 (derived ASD and standard deviation of the distribution). They indicate that even at the lowest concentration ∼20 mmol·L^–1^ (being identical to the ionic strength in this case: see caption of Figure 4) the ASD of the obtained PMMA spheres (∼440 nm) is distinctly larger as compared to the standard synthesis without added salt—factually by a factor of almost 2.

**Figure 3:**
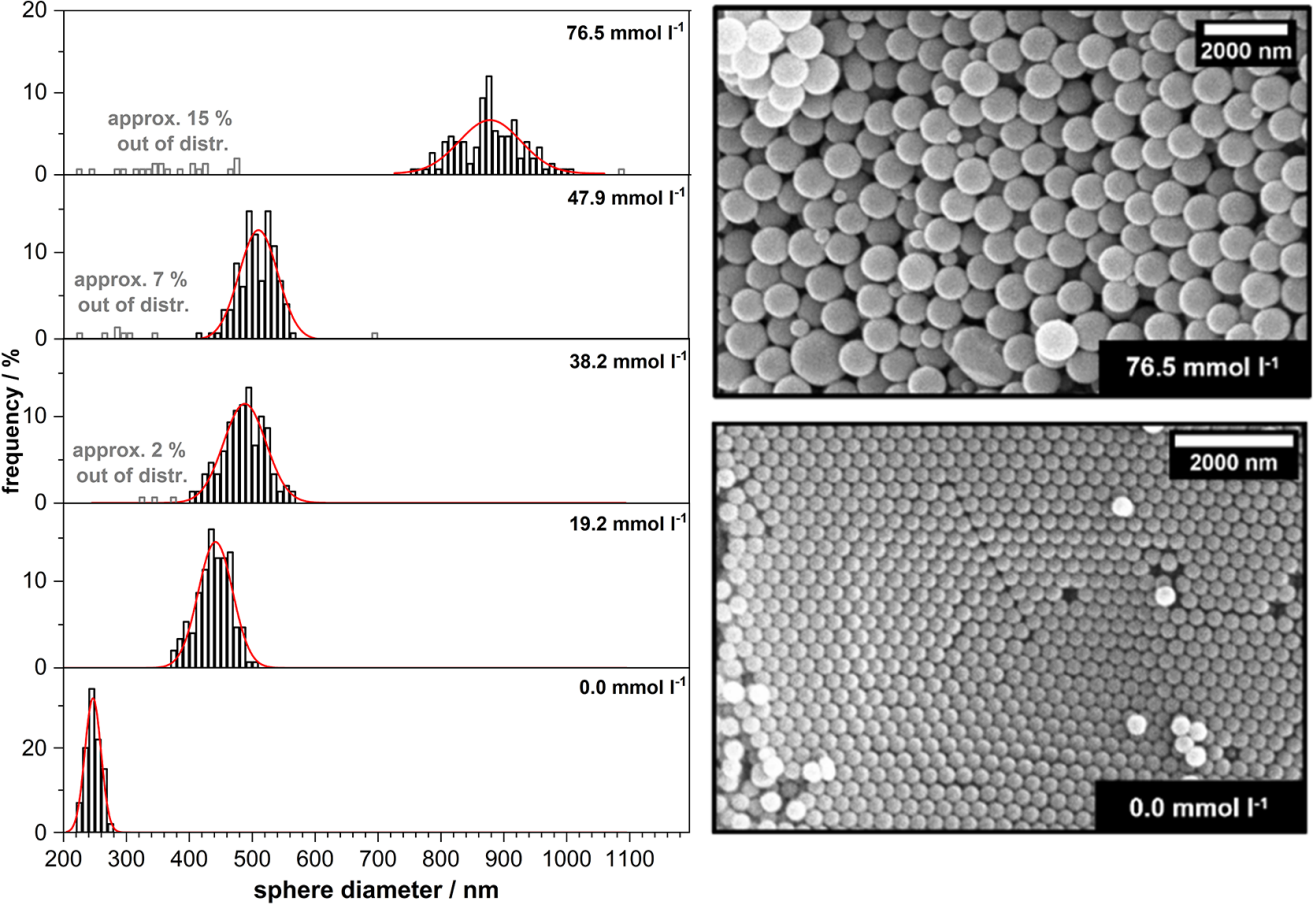
Distribution of PMMA sphere diameters (left) obtained for increasing concentrations of NaCl added to the emulsion (0, 19.2, 38.2, 47.9, and 76.5 mmol·L^–1^) before starting the polymerization reaction at 383 K. The red lines represent the fitted Gaussian distribution function for each histogram. On the right-hand-side SEM images of the close-packed sphere arrangements obtained after drying are shown for the lowest (0 mmol·L^–1^) and highest NaCl concentration (76.5 mmol·L^–1^).

**Table 1:** Arithmetic Average of the Size Distribution (ASD) and Two-Sigma Standard Deviation (2σ) of PMMA Spheres as a Function of the NaCl Concentration Added

[NaCl]/mmol L^–1^	ASD/nm	2σ/%
0.0	257	15.4
19.2	439	11.8
28.7	426	9.2
38.3	483	16.3
47.9	502	12.7
57.5	561	12.4
67.1	628	14.0
76.5	804	11.9

A combination of two factors is likely to induce this effect. On the one hand, the added ions reduce the solubility of the MMA monomers in the water phase even further, meaning that fewer oligomers grow at the same time and compete for available monomers released from the droplets. Accordingly, longer chains form, which, upon aggregation, also form larger seed particles and hence larger spheres after completing the second (physical) growth phase.^12,13^ On the other hand, electrostatic repulsion between the particles, resulting from their positive zeta potential and, in the absence of an electrolyte, hindering their coalescence in the second phase, is reduced in the presence of a salt. In this case, its anions—here chloride—can partially neutralize the positive surface charge,^22^ hence facilitating the fusion of the primary seed particles to larger entities by coalescence under otherwise identical synthesis conditions.

The development of the sphere diameters (ASDs) upon raising the salt concentration to even higher values is depicted in Figure 4. The exponential increase of the ASD as a function of the ionic strength I demonstrates that the former can be almost doubled if the latter is quadrupled. As inferable from Figure 3, however, this strategy is associated also with a certain problem. While for concentrations higher than ∼20 mmol·L^–1^, the majority of spheres still exhibit diameters in accordance with a Gaussian distribution that is similarly narrow than the one found at low ionic strength, also some outliers with significantly smaller sizes are detectable. Noticeably, their share becomes larger as the ionic strength increases, reaching a value of about 15% at the highest investigated concentration (∼75 mmol·L^–1^).

**Figure 4:**
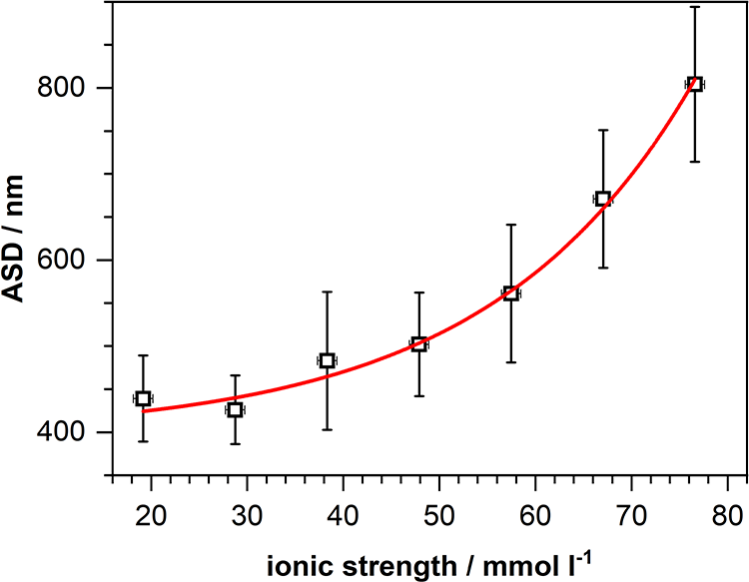
Arithmetic average sphere diameter (ASD) of PMMA spheres synthesized at 383 K as a function of the ionic strength I of the water phase after adding NaCl in increasing concentrations. Note that *I* is defined as $I=\frac{1}{2}\sum_{i}c_{i}\cdot z_{i}^{2}$ with *c*_*i*_: molar concentration of ion i in the solution; *z*_*i*_: charge of this ion. In case of NaCl the charges are +1 and −1, resulting in an ion strength *I* which equals the molar concentration of NaCl added.

At present, the reason for this behavior is not clear. It may be speculated, though, that the declining monomer concentration in the aqueous phase upon raising the NaCl concentration results in spatial inhomogeneities and thus growth kinetics in the emulsion, finally leading to the outliers not fitting into the Gaussian distribution.

Up to a concentration of 40 mmol·L^–1^ the sphere size distribution (ASD ∼ 450 nm) is still uniform enough so that, upon drying, well-ordered close-packed sphere arrangements are obtainable which can be directly employed as templates for inverse opal structures. Beyond that value, the increasing fraction of spheres not fitting into the range of normally distributed sizes prevent their ordering. In case, however, this perturbing part can be separated and removed, e.g., by centrifugation, this problem may be overcome.

In essence, the findings clearly show that the addition of salts and the variation of the ionic strength represent a viable synthetic pathway to prepare PMMA spheres with ASDs in a large range between 250 and 800 nm. Furthermore, smaller diameters down to 170 nm are assessable without a cosolved salt (see previous subsection), just by increasing the synthesis temperature to about 430 K.

### 3.3 Opalescence Properties

To finally check the quality of the close-packed sphere layers, obtained after coating and drying the final dispersions on a planar surface, and, in this way, to verify their suitability as templates for inverse opal structures, diffuse reflectance UV/vis spectroscopic measurements were carried out for the range up to ∼300 nm. Well-ordered films with a high periodicity will interfere with electromagnetic radiation when sphere diameters and the wavelength lie in a similar length regime.^23,24^ As a consequence, this wavelength is then reflected—a phenomenon called opalescence effect.^7^ On this basis, a linear relationship between the ASD and the position of the maximum seen in UV/vis reflectance spectra is expected.

In Figure 5 the experimental results are presented. In agreement with the prediction indeed a linear correlation is given over the entire size range. This finding once again proves the uniformity of the prepared PMMA spheres and the resulting high periodicity of the layers fabricated from them, hence qualifying these systems as excellent templates for the manufacture of inverse opal structures.

**Figure 5:**
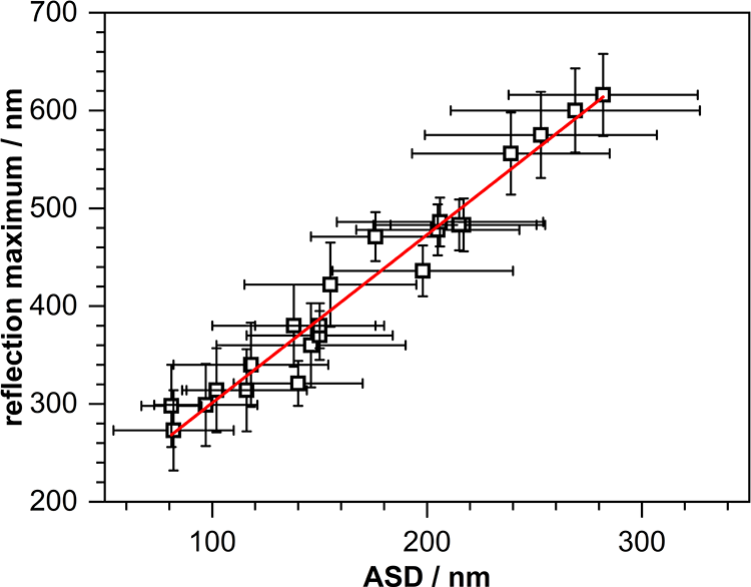
Dependence of the wavelength maximum detected in diffuse reflectance UV/vis spectra recorded for coated PMMA spheres with different sizes (ASD). Red line: linear fit (λ_max_/nm = 1.72·ASD/nm + 122).

## 4 Conclusion

Uniform PMMA spheres could be successfully synthesized in a diameter range of 170–800 nm by a simple emulsion polymerization process synthesis route needing no emulsifier (surfactant). Since the synthesis is carried out under reflux conditions, a protective gas atmosphere is dispensable as well. The growth of the polymer spheres was shown to mechanistically consist of two steps. In a first stage seed particles emerge as a result of oligomer formation and aggregation in the aqueous phase. During a second stage these entities successively coalesce resulting in spherical particles with a diameter approximately double as large as the primary particles.

In the temperature range below 400 K spheres with a diameter of about 250 nm are obtained. Above this temperature the decomposition of the used initiator gets accelerated and the diameter decreases to 170–180 nm. Around 400 K a small temperature window exists in which intermediate sizes are assessable. A distinctly better way to tune the particle sizes; however, is provided when adding a salt, such as NaCl, to the starting emulsion. With increasing concentrations, i.e., with increasing ionic strength of the water phase, the sphere diameters can be pushed to values being about 3-times larger as compared to syntheses without a salt, while still exhibiting a comparatively narrow size distribution. When coating and drying the spheres on a planar support, well-ordered close-packed layers with long-range periodicity emerge. These may be subsequently used to fabricate inverse opal structures with tailored optical properties.

## Supplementary Material



## Notes

Overview of the determined spheres sizes and their errors of the different time- and temperature-dependent experiments. Additionally, SEM micrographs of dried films of temperature-dependent experiments for PMMA spheres synthesized at selected temperatures of 363, 383, 413 and 433 K and their corresponding histograms are shown (PDF)
