# Supplementary material for: Tailormade PMMA Spheres: Synthesis and Growth Mechanism
Source: ACS Omega. 2025 Jun 4;10(23):24359–66. doi: 10.1021/acsomega.5c00402 (PMC12177621; doi:10.1021/acsomega.5c00402)
Supplement: Supplementary file 1 [file ao5c00402_si_001.pdf]

Supporting information file for:
Tailormade PMMA spheres: synthesis and growth mechanism

Oliver Thüringer[1,2], Raphaell Moreira[2], Marcus Bäumer[2,3]*, Cecilia B. Mendive[4], Thorsten M. Gesing[1,3], Alexander Wollbrink[1,3]

* Corresponding author: Marcus Bäumer. Email: baeumer@uni-bremen.de

[1]University of Bremen, Institute of Inorganic Chemistry and Crystallography, Leobener Straße 7, 28359 Bremen, Germany

[2]University of Bremen, Institute of Applied and Physical Chemistry, Leobener Straße 6, 28359 Bremen, Germany

[3]University of Bremen, MAPEX Center for Materials and Processes, Bibliothekstraße 1,

28359 Bremen, Germany

[4]Universidad Nacional de Mar del Plata, Facultad de Ciencias Exactas y Naturales, Departamento de Química, Funes 3350, B7602AYL Mar del Plata, Argentina

Table S1 Arithmetic average of sphere size distribution (ASD), two-sigma standard deviation (2σ) and relative sphere growth (RSG) of PMMA spheres determined by SEM of time-dependent experiments for PMMA spheres synthesized at selected temperatures

	Synthesis temperature					
	363 K		393 K		433 K	
time /s	ASD /nm	2σ /%	ASD /nm	2σ /%	ASD /nm	2σ /%
120						
180					139	21.4
240					141	20.0
300					140	20.0
360			82	20.0	149	20.0
420			97	18.0		
480			102	27.3		
540			116	26.6		
600			150	22.7		
660	81	11.0	198	22.0		
720	118	22.2	176	16.7		
780	146	18.3	205	21.9		
840	138	32.7	217	19.0		
900	155	25.0	215	9.5		
960	239	19.1	193	12.4		
1020	253	20.0				
1080	253	12.1				
1140	276	21.0				
1200	269	16.4				
1260	282	17.0				

Table S2 Arithmetic average of sphere size distribution (ASD) of PMMA spheres determined by SEM and the corresponding two-sigma standard deviation (2σ) of calculated ASD.

Temperature /K	ASD /nm	2σ /%
363	257	15.4
373	243	8.3
383	247	8.0
393	205	14.3
403	185	15.8
413	183	22.3
423	183	16.7
433	171	11.8

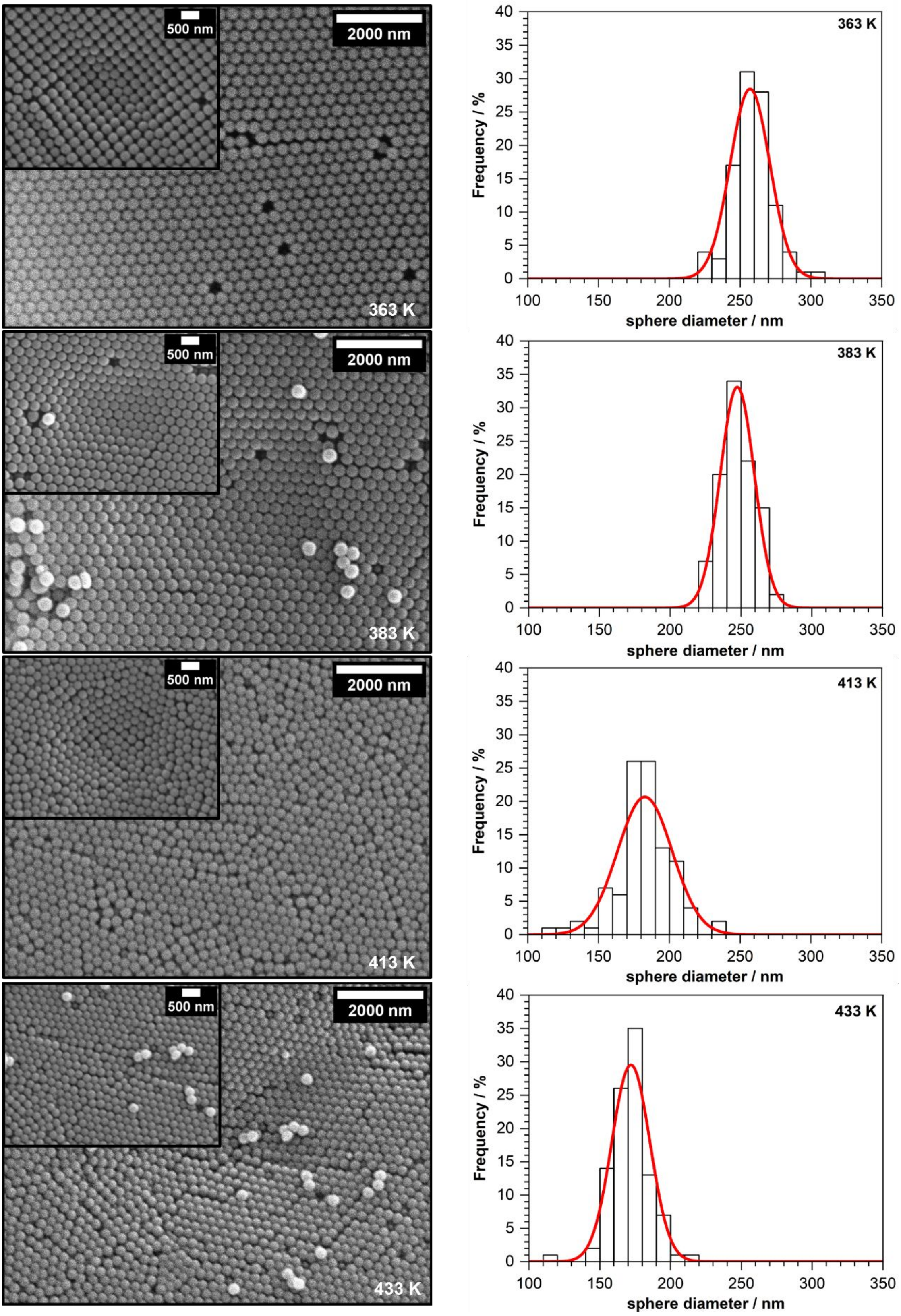

Figure S1 SEM micrographs of dried films of temperature-dependent experiments for PMMA spheres synthesized at selected temperatures of 363 K, 383 K, 413 K and 433 K (left). The corresponding histograms show the sphere size distribution (SSD) for selected temperatures (right). The red lines show the calculated normal distribution of each histogram.
